# Real-time PCR for *Francisella tularensis* Types A and B

**DOI:** 10.3201/eid1211.060629

**Published:** 2006-11

**Authors:** Kiersten J. Kugeler, Ryan Pappert, Yan Zhou, Jeannine M. Petersen

**Affiliations:** * Centers for Disease Control and Prevention, Fort Collins, Colorado, USA

**Keywords:** Francisella tularensis, tularemia, real-time PCR, type A, type B, letter

To the Editor: Francisella tularensis, the etiologic agent of tularemia, is highly infectious and considered a potential bioweapon (*1**–**3*). Although 4 subspecies of F. tularensis are recognized, most cases of tularemia are due to infection by subsp. tularensis (type A) or holarctica (type B). North America is the only region where both type A and type B cause human disease. Subspecies novicida is also found in North America, but it is of reduced virulence. Disease incidence attributable to either type A or type B is essentially unknown because the traditional method for classification of these subspecies is glycerol fermentation, which requires culture recovery (*4*). F. tularensis is fastidious and slow growing, with isolates recovered in a small percentage of cases.

We developed real-time TaqMan PCR assays for classification of F. tularensis type A and type B after F. tularensis is identified by culture or, in the absence of culture, by a PCR method such as the F. tularensis multitarget TaqMan assay (*5*). The type A TaqMan assay targets pdpD, which is present in type A, almost entirely absent from type B, and contains a 144-bp insert in novicida (*6**,**7*) (F: 5´-GAGACATCAATTAAAAGAAGCAATACCTT-3´; R: 5´-CCAAGAGTACTATTTCCGGTTGGT-3´; probe: 5´-AAAATTCTGC"T"CAGCAGGATTTTGATTTGGTT-3´). The type B assay targets a junction between ISFtu2 and a flanking 3´ region (GenBank AY06) (F: 5´- CTTGTACTTTTATTTGGCTACTGAGAAACT-3´; R: 5´- CTTGCTTGGTTTGTAAATATAGTGGAA-3´; probe: 5´- ACCTAGTTCAACC"T"CAAGACTTTTAGTAATGGGAATGTCA-3´). In type A and novicida, ISFtu2 is absent from this position (*8*). Oligonucleotides were designed with Primer Express version 2.0 (Applied Biosystems, Foster City, CA, USA). Probes were synthesized with a 5´ 6-carboxy-fluorescein reporter and an internal quencher (either BHQ1 [type A] or QSY-7[type B]) at the nucleotide position indicated by the quotation marks.

Assays were optimized by using 1 ng of type A (strain SchuS4) or type B (strain LVS) DNA on the LightCycler 1.2 (Roche Applied Science, Indianapolis, IN, USA). Optimized concentrations (20 μL final volume) were 1× LightCycler Fast Start DNA Master Hybridization Probe mix (Roche), 750 nmol/L primers, 200 nmol/L probe, 5 mmol/L MgCl_2_ and 0.5 U uracil-DNA glycosylase. PCR conditions were 50°C for 2 min, 95°C for 10 min, 45 cycles of 95°C for 10 s and 65°C for 30 s, then 45°C for 5 min. Cycle threshold (C_t_) values were calculated by using the second derivative maximum method with the y-axis at F1/F3 (LightCycler software version 3.5).

Sensitivity of each assay was assessed by using 10-fold serial dilutions (100,000 to 1 genomic equivalents [GE]) of SchuS4 or LVS DNA. Testing was performed in triplicate, with a reproducible detection limit of 10 GE for both assays. Specificity of each assay was tested with 1 ng of DNA from a panel of 62 Francisella isolates (Table A1) and 22 non-Francisella isolates (Acinetobacter, Bacillus, Brucella, Corynebacterium, Enterobacter, Enterococcus, Escherichia, Haemophilus, Klebsiella, Legionella, Proteus, Pseudomonas, Serratia, Staphylococcus, Streptococcus, and Yersinia species). Isolates were grown, DNA purified, and quantified as previously described (*5*). Specificity was also evaluated with DNA (2 μL) extracted as previously described from Francisella-like tick endosymbionts of Dermacentor variabilis and Francisella-like soil bacteria (Table A1) (*9**,**10*). The type A assay recognized all type A isolates with an average C_t_ value of 17.9 (n = 19). The type B assay detected all type B strains with an average C_t_ value of 17.1 (n = 21). Neither assay displayed cross-reactivity with F. tularensis subsp. novicida (n = 7), F. philomiragia (n = 15), Francisella-like tick endosymbionts (n = 3), Francisella-like soil bacteria (n = 7) (Table A1), or non-Francisella spp. (n = 22).

To evaluate the ability of the type A and type B TaqMan assays, in conjunction with the multitarget assay, to identify F. tularensis and classify subspecies in primary specimens, human, animal, and tick samples were tested (Table ). DNA was extracted from 200 μL fluid, 25 mg liver, and 10 mg spleen or lung by using the QIAamp DNA MiniKit (Qiagen, Valencia, CA, USA) and 1 μL tested. Multitarget PCR conditions were as described (*5*).

The multitarget and subspecies-specific PCR assays accurately identified and classified F. tularensis in all specimens positive by standard diagnostic methods (Table). In addition, the type A and type B assays provided subspecies information for positive specimens in which an isolate was not recovered for glycerol fermentation testing (Table ). All specimens negative by standard diagnostic methods tested negative by PCR. These preliminary results suggest that a F. tularensis PCR identification method, in combination with the type A and type B assays, provides the capability to identify F. tularensis and determine subspecies in the absence of culture.

We describe real-time PCR assays capable of classifying F. tularensis type A and type B and distinguishing these subspecies from the less virulent subsp. novicida. These assays are designed for use after F. tularensis has been identified by culture or by PCR. Supplemental use of these assays will allow laboratories to actively subtype F. tularensis isolates and primary specimens, thus providing subspecies information for a higher percentage of tularemia cases. Improved subspecies information will further understanding of the disease incidence and geographic distribution of F. tularensis type A and type B in North America.

**Table:** Comparison of standard diagnostic methods with the multitarget *Francisella tularensis* TaqMan assay and type A and type B assays using primary specimens

Specimen	Source	*F. tularensis* identified*	Subspecies identification†	Multitarget *F. tularensis* TaqMan assay‡			Type A assay‡ (C_t_ value)§	Type B assay‡ (C_t_ value)
				IS*Ftu2*	*IglC*	*tul4*		
Lymph node aspirate	Human	+		+	+	+	31	–
Bronchial wash	Human	+	A	+	+	+	34	–
Upper lung	Human	+	A	+	+	+	20	–
Lower lung	Human	+	A	+	+	+	26	–
Liver	Human	+	A	+	+	+	29	–
Spleen	Human	+	A	+	+	+	31	–
Pleural fluid	Human	+	B	+	+	+	–	36
Blood	Human	+		+	+	+	–	38
Spleen	Human	–		–	–	–	–	–
Liver	Human	–		–	–	–	–	–
Cerebrospinal fluid	Human	–		–	–	–	–	–
Blood	Human	–		–	–	–	–	–
Liver/spleen	Tamarin	+		+	+	+	28	–
Tissue	Tick¶	+	A	+	+	+	26	–
Tissue	Tick¶	+	A	+	+	+	33	–
Blood	Prairie dog	+	B	+	+	+	–	30
Blood	Prairie dog	+	B	+	+	+	–	27
Spleen	Prairie dog	+	B	+	+	+	–	21
Spleen	Prairie dog	+	B	+	+	+	–	31
Spleen	Prairie dog	–		–	–	–	–	–
Liver	Cat	–		–	–	–	–	–
Liver	Rat	–		–	–	–	–	–
Spleen	Rat	–		–	–	–	–	–
Spleen	Squirrel	–		–	–	–	–	–

*F. tularensis infection identified by culture, direct fluorescent antibody testing, or serologic testing. †Subspecies was determined by glycerol fermentation when an isolate was recovered. ‡+ = positive result, 17<C_t_ <38; – = negative result, no fluorescence detected after 45 cycles of amplification. §C_t_, cycle threshold. ¶Tick species tested were *Haemaphysalis leporispalustris* and *Dermacentor andersoni*.

## Appendix

**Table A1:** Specificity evaluation with DNA from Francisella spp

Organism	Sample ID	Source	Geographic origin	Type A assay (C_t_ value)*	Type B assay (C_t_ value)*
F. tularensis subsp. tularensis (type A)					
	SchuS4	Human	Ohio	15.9	–
	WY963418	Human	Wyoming	15.9	–
	MA002987	Human	Massachusetts	18.4	–
	CO012364	Cat	Colorado	18.3	–
	CO013713	Rabbit	Colorado	16.5	–
	ND000952	Human	North Dakota	16.9	–
	KS000948	Cat	Kansas	16.7	–
	OK002731	Human	Oklahoma	18.4	–
	NC993990	Rabbit	North Carolina	16.8	–
	AR000028	Human	Arkansas	18.8	–
	UT983134	Human	Utah	16.9	–
	NM990295	Rabbit	New Mexico	17.2	–
	NC973057	Rabbit	North Carolina	16.5	–
	NC015379	Cat	North Carolina	18.6	–
	AR982146	Rabbit	Arkansas	18.0	–
	OK004337	Human	Oklahoma	18.4	–
	AR011117	Human	Arkansas	22.9	–
	MO011907	Human	Missouri	20.3	–
	CA020099	Human	California	18.2	–
F. tularensis subsp. holarctica (type B)					
	LVS	Rat	Russia	–	16.0
	KY993387	Human	Kentucky	–	19.7
	OR960246	Monkey	Oregon	–	16.7
	CN985979	Human	Canada	–	17.1
	AZ001325	Rat	Arizona	–	16.2
	IL003633	Human	Illinois	–	18.4
	MO011673	Human	Missouri	–	16.8
	KY001708	Human	Kentucky	–	15.8
	OH013029	Prairie dog	Ohio	–	16.7
	UT002098	Human	Missouri	–	18.2
	SP986120	Rabbit	Spain	–	17.5
	IN983055	Rat	Indiana	–	15.6
	CO961243	Vole	Colorado	–	15.5
	CA990837	Human	California	–	16.5
	IN002758	Human	Indiana	–	15.8
	AZ001324	Squirrel	Arizona	–	15.9
	CA993992	Monkey	California	–	16.9
	SP982108	Human	Spain	–	17.8
	NM002642	Human	New Mexico	–	17.9
	JAP5-3-11	Human	Japan	–	18.6
	KO971026	Human	Korea	–	19.0
F. tularensis subsp. novicida					
	GA993548	Human	Louisiana	–	–
	GA993549	Human	California	–	–
	GA993550	Water	Utah	–	–
	UT014992	Human	Utah	–	–
	AS020814	Human	Australia	–	–
	FX1	Human	Texas	–	–
	FX2	Human	Texas	–	–
F. philomiragia					
	GA012793	Human	California	–	–
	GA012794	Human	Colorado	–	–
	GA012795	Human	New York	–	–
	GA012796	Human	California	–	–
	GA012797	Human	Pennsylvania	–	–
	GA012799	Human	Connecticut	–	–
	GA012800	Human	Connecticut	–	–
	GA012801	Human	New York	–	–
	GA012802	Human	California	–	–
	GA012803	Human	New Mexico	–	–
	GA012804	Human	Virginia	–	–
	GA012806	Human	Massachusetts	–	–
	GA012810	Water	Utah	–	–
	GA012811	Water	Utah	–	–
	ATCC 25015	Muskrat	Utah	–	–
Francisella-like tick endosymbionts†					
	2040372	Tick	California	–	–
	2040460	Tick	California	–	–
	MV2	Tick	Massachusetts	–	–
Francisella-like bacteria‡					
	005	Soil	Texas	–	–
	013	Soil	Texas	–	–
	015	Soil	Texas	–	–
	027	Soil	Texas	–	–
	045	Soil	Texas	–	–
	034	Soil	Texas	–	–
	039	Soil	Texas	–	–

*C_t_, cycle threshold; –, negative result, no fluorescence detected after 45 cycles of amplification. †See Kugeler et al. (*10*). ‡See Barns et al (*9*).
